# Health-Related Quality of Life and Associated Factors in Patients with Heart Failure Across Three Ejection Fraction Categories: A Two-Center Cross-Sectional Study

**DOI:** 10.3390/healthcare14162447

**Published:** 2026-08-07

**Authors:** Osama Alkouri, Walid Al-Qerem, Yousef Khader, Abdulkareem Alshehri, Ahmad M. Al-Bashaireh, Ghaleb Alharbi, Jolly Isaac, Eidan Alrasheid, Nezam Al-Nsair, Mohannad Alkhateeb, Nizar Alsubahi

**Affiliations:** 1 Faculty of Nursing, Yarmouk University, Irbid 21110, Jordan; 2 Faculty of Pharmacy, Al-Zaytoonah University of Jordan, Amman 11733, Jordan; 3 Department of Public Health, Faculty of Medicine, Jordan University of Science and Technology, Irbid 21110, Jordan; 4 Advanced Diagnostic and Therapeutic Institute, King Abdulaziz City for Science and Technology (KACST), Riyadh 12354, Saudi Arabia; 5 Faculty of Nursing, Philadelphia University, Amman 19392, Jordan; 6 Department of Clinical Pharmacy, College of Pharmacy, Shaqra University, Shaqra 11961, Saudi Arabia; 7 Hind Bint Maktoum College of Nursing and Midwifery, Mohammed Bin Rashid University of Medicine and Health Sciences, Dubai Health, Dubai 505055, United Arab Emirates; 8 Nursing Department, Ministry of Health, Kuwait City 13001, Kuwait; 9 Department of Health Service and Hospital Administration, Faculty of Economics and Administration, King Abdulaziz University, Jeddah 21589, Saudi Arabia; 10 School of Public Health and Community Medicine, Institute of Medicine, University of Gothenburg, 40530 Gothenburg, Sweden; 11 Department of Health Services Research, Care and Public Health Research Institute (CAPHRI), Maastricht University Medical Center, Faculty of Health, Medicine and Life Sciences, Maastricht University, 6200 MD Maastricht, The Netherlands

**Keywords:** heart failure, health-related quality of life, ejection fraction, anxiety, depression, self-care confidence

## Abstract

**Background:** Heart failure (HF) substantially affects health-related quality of life (HRQoL), but evidence across left ventricular ejection fraction (LVEF) categories in Jordan remains limited. **Methods:** This multicenter cross-sectional study included 516 adults with HF attending outpatient cardiac clinics in northern Jordan between May and November 2025. SF-36 Version 1.0 items were recoded to eight 0–100 domains, and unweighted SF-36 physical and mental four-domain composite scores were analyzed using expanded multivariable ordinary least-squares models with HC3 robust standard errors. A total of 476 participants had complete data for all model variables. **Results:** The mean SF-36 physical and mental four-domain composite scores were 48.80 (SD 21.14) and 51.88 (SD 20.57), respectively. Both scores differed across EF categories before adjustment (physical *p* < 0.001; mental *p* < 0.001), but the adjusted EF omnibus tests were not significant (physical *p* = 0.111; mental *p* = 0.613). Lower physical scores were associated with NYHA class II and III, physical inactivity, sleeping difficulty, retirement, and higher anxiety and depression scores, whereas self-care efficacy was positively associated with physical HRQoL. Lower mental scores were associated with NYHA class II and III, physical inactivity, and higher anxiety and depression scores, while self-care efficacy was positively associated with mental HRQoL. **Conclusions:** In this cross-sectional sample, functional limitation, anxiety and depressive symptoms, physical inactivity, and self-care efficacy were more consistently associated with HRQoL than EF category after adjustment. These findings support multidimensional assessment alongside cardiac measures.

## 1. Introduction

Heart failure (HF) is one of the most prevalent chronic diseases, impacting approximately 1% to 2% of adults [1,2,3]. Studies reported that nearly 64.3 million people are diagnosed with HF worldwide [4,5]. Despite advancements in treatment procedures, the rates of morbidity, mortality, and healthcare costs associated with heart failure continue to be high [4]. Recent studies have shown that 25% of heart failure patients hospitalized are readmitted within 30 days of discharge, while 50% are readmitted within 6 months [6,7]. Furthermore, heart failure is responsible for nearly 8.7% of all mortalities attributed to cardiovascular disease in Jordan [8].

Developing countries, such as Jordan, have witnessed a noticeable increase in cases diagnosed with heart failure [9]. The prognosis for heart failure in the Middle Eastern countries is alarming, with an estimated 25% mortality rate within the first year of diagnosis and 50% within five years [10,11]. In Jordan, HF is a widespread cardiovascular condition leading to increased death rates and serious health complications in people [11]. A study from Jordan showed that around 100,000 people in the country are living with heart failure, with about 8300 new cases diagnosed each year [12].

Jordan is undergoing a demographic shift, as the number of people aged 60 years and above is projected to more than double between 2020 and 2050, rising from around 6% (620,000 cases) to 16.6% (2.15 million) of the total population. This age group is considered a major risk factor for developing other comorbidities such as hypertension, diabetes, and hyperlipidemia, which are significant contributing factors for the development of heart failure [13]. These HF-related risk factors are especially prevalent in Jordan and exert a significant health burden on the country, accounting for nearly 42% of all deaths. A national STEPwise survey (STEPs), in collaboration with the World Health Organization (WHO) and the United States Agency for International Development (USAID), indicates that the prevalence of hypertension in Jordan is 22%, with nearly half (47.8%) of those affected not taking medication. Diabetes affects 20% of people aged 45–69 years, and hypercholesterolemia is prevalent in 17.7% of the population [14]. Additionally, the survey indicates that 41% of people in Jordan are current tobacco smokers. Notably, 24.5% of the survey population is either currently suffering from cardiovascular diseases or at high risk of developing them within the next 10 years [14].

HF is often associated with various symptoms such as fatigue, edema, shortness of breath, exercise intolerance, decreased muscle strength, and low weekly physical activity, which may occur individually or in combination [15,16,17]. Due to the progressive nature of heart failure, its symptoms tend to deteriorate or aggravate over time, which ultimately increases difficulty for patients to carry out daily activities [16,18].

The effects of HF surpass physical restrictions, significantly impacting the psychological, mental, social, emotional, and economic well-being of patients, eventually reducing their overall quality of life (QoL) [17]. Research has demonstrated that people living with HF experience a poorer quality of life compared to the general population and to patients with other chronic diseases [17,19,20].

The World Health Organization (WHO) defines QoL as a person’s perception of their life position, considering the cultural and value systems they belong to, as well as their standards, goals, expectations, and concerns [21,22].

The concept of health-related QoL refers to patients’ perception of how their clinical conditions and treatments affect their lives [22,23]. Particularly, it assesses physical, social, psychological, emotional, and cognitive functioning [21,22]. Poor quality of life is consistently associated with patients’ outcomes related to the HF condition, such as increased deaths and hospitalizations, as well as low adherence to self-care [21,24,25].

Many factors are associated with the health-related QOL of people with HF. Age, level of education, gender, having other comorbidities, smoking, living status, place of residence, functional class of HF, and self-care behavior were found to be associated with QoL [21,26]. Previous studies have identified diverse factors associated with poorer QoL in patients with HF, including female gender, young age, being single, low educational level and income, existence of depression, low ventricular ejection, high New York Heart Association (NYHA) functional class, exposure to tobacco, history of hospitalization, polypharmacy, comorbidity, poor adherence to medication, insufficient self-care, and heart failure duration [27,28,29,30].

However, several systematic reviews [11,21,31,32] found the following: (1) studies examining factors associated with QOL in people with HF remain limited, particularly in Middle Eastern countries such as Jordan [33,34]; and (2) although associations between clinical factors and QOL may vary across the left ventricular ejection fraction (LVEF) spectrum, studies focusing on specific subgroups within the HF population are lacking [35]. Identifying QOL levels and the factors associated with them in adults with HF is essential for tailoring personalized interventions that align with patients’ needs and priorities. This approach can inform the development of patient-centered strategies, address disparities, improve monitoring and management across diverse HF subpopulations, enhance patient outcomes, and ultimately prevent more severe health-related complications [20,36]. Thus, this study was conducted to identify QOL levels and the factors associated with them among Jordanians with HF who had reduced, mildly reduced, or preserved ejection fraction.

The theoretical framework upon which this research rests is informed by the WHO’s definition of quality of life and Wilson & Cleary’s Health-Related Quality of Life Model [37] in which biological, clinical, functional, and environmental factors are considered in relation to patient well-being. In the current study, the theoretical framework has been adapted to take into consideration the case of heart failure patients in Jordan, where each component of the framework is divided into three domains of factors associated with HRQoL. Sociodemographic factors constitute the first domain where age, gender, education, economic status, marital status, and area of residency are taken into consideration because of their relationships with access to health services, health literacy, and available social support. Clinical factors are included in the second domain, where NYHA functional class, LVEF category, HF duration, co-morbid conditions (hypertension, diabetes mellitus, hyperlipidemia), polypharmacy, hospitalization, and smoking are considered among others. The last domain focuses on psychosocial and behavioral factors, where depression, anxiety, medication adherence, self-care practices, social support, exercise, and symptoms are among the key factors [38].

## 2. Methods

### 2.1. Study Design and Settings

A cross-sectional survey was distributed to a consecutive sample of patients with HF who regularly visit cardiac clinics at two hospitals in northern Jordan. The study was conducted between May and November 2025.

### 2.2. Ethical Considerations

This study was conducted between May and November 2025 in accordance with the Declaration of Helsinki and received ethical approval from the Institutional Review Boards of King Abdullah University Hospital and Princess Basma Hospital (Approval ID: Apr2025/181-21). Permission to access outpatient clinics and medical records was obtained from hospital administrations. All participants provided written informed consent after receiving information about the purpose of the study, procedures, and their rights. Participation was voluntary, and confidentiality was maintained through coded identifiers and secure data storage accessible only to the research team.

### 2.3. Inclusion and Exclusion Criteria

The inclusion criteria for this study were patients aged 18 years or older who had been diagnosed with heart failure (HF) by a cardiologist using echocardiography, EF measurements, NYHA classification, and HF signs and symptoms. The exclusion criteria included individuals who were unable to complete the survey due to severe dementia. Illiterate participants were allowed if they had a consenting companion who could assist with completing the survey. Patients with cognitive impairments, such as dementia or psychosis, who may not recall their conditions accurately, as well as those in NYHA stage IV, were excluded. Additionally, participants with acute conditions, such as acute HF exacerbation, severe chest infections, or any infection likely to significantly impact their quality of life, were excluded. Patients with coexisting chronic illnesses, including chronic obstructive pulmonary disease, end-stage renal disease, chronic liver disease, and cerebrovascular accidents, were not included in the study as well.

### 2.4. Sample Size

An a priori sample-size calculation was conducted for an omnibus fixed-model multiple linear regression based on the original model specification. Assuming 29 predictor degrees of freedom, a medium effect size (Cohen’s f^2^ = 0.15), α = 0.05, and 80% power, the minimum required sample was 184 participants [39]. The final recruited sample comprised 516 participants, of whom 476 had complete data for the primary analyses.

### 2.5. Data Collection Procedure

Data collection commenced after ethics approval was obtained. Outpatient clinic administrators were then approached for permission to conduct the study, with assurances that patient safety would be ensured and staff workload minimized. The administrator referred the researcher to a physician who works with the cardiologist and has access to patient records. This physician identified eligible patients and shared the list with the nurses. The nurse then informed eligible patients about the study during their appointment.

The participants of interest, those agreeing to participate in the study, were contacted by the researcher, who introduced himself, discussed the aims and a conceptual understanding of the potential advantages, and provided a means of contacting him with questions. It was made known to those agreeing to participate in the research that there would be five questionnaires in total, which would cumulatively take approximately 50 min to complete. For participants who could not complete a questionnaire themselves due to illiteracy, assistance was provided by a family member or friend. The clinic nurse noted several variables for each participant; these included BMI, EF, functional classification according to the New York Heart Failure classification, and other considerations specifically related to heart failure.

### 2.6. Participant Recruitment

**Figure 1:**
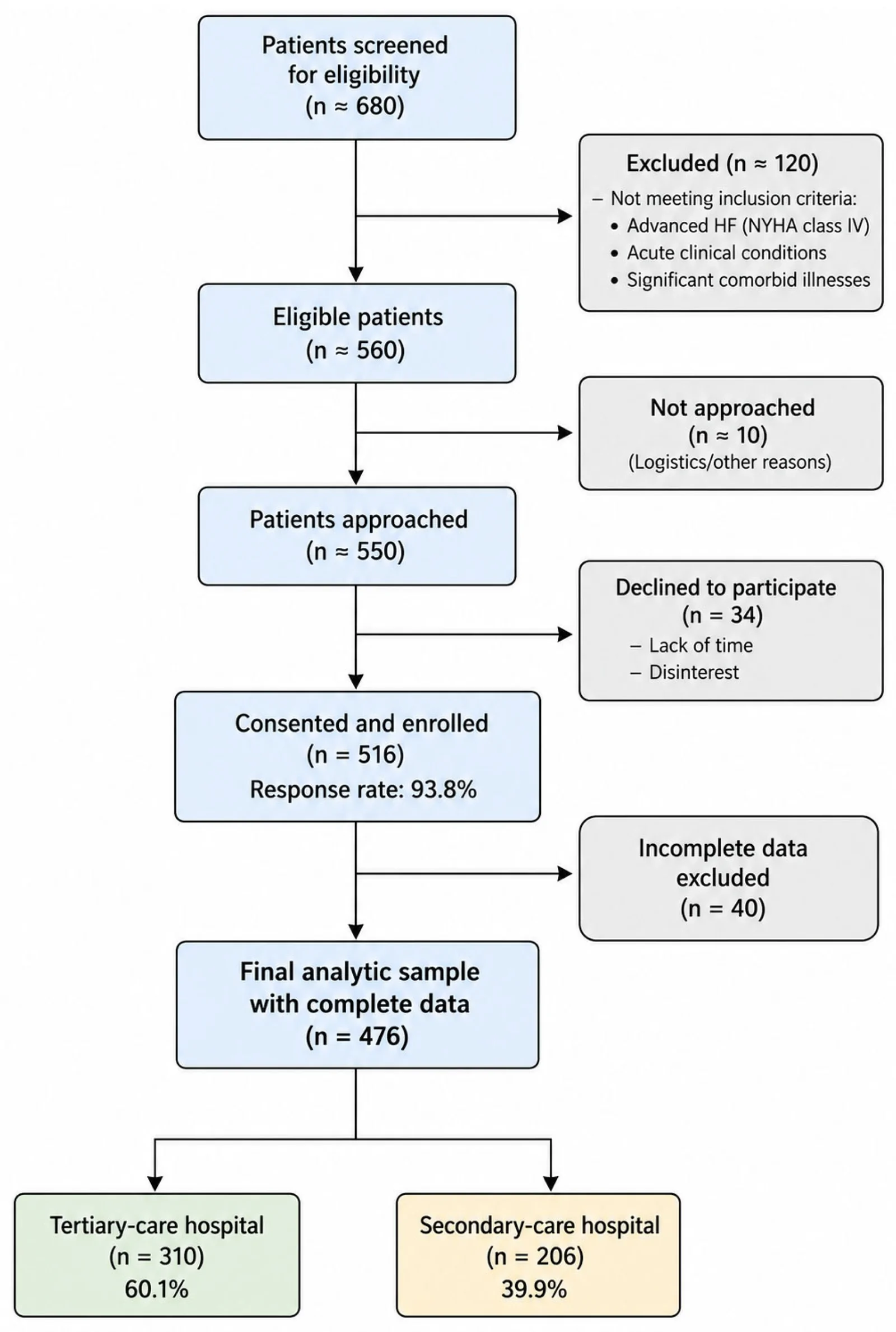
Participant flow diagram in accordance with STROBE guidelines.

As shown in Figure 1, a total of 680 patients with heart failure were screened for eligibility across the two participating hospitals. Of these, 120 patients were excluded due to not meeting the inclusion criteria, including advanced heart failure (NYHA class IV), acute clinical conditions, or significant comorbid illnesses. A total of 560 patients were eligible, of whom 550 were approached to participate in the study. Among those approached, 34 patients declined participation, primarily due to lack of time or disinterest. A total of 516 patients consented and were enrolled, yielding a response rate of 93.8% (516/550).

Among enrolled participants, 40 individuals provided incomplete data and were excluded from the final regression analyses. Therefore, the final analytic sample consisted of 476 participants with complete data. Recruitment was conducted across two hospitals, with 310 participants (60.1%) enrolled from the tertiary-care hospital and 206 participants (39.9%) from the secondary-care hospital.

### 2.7. Data Collection Tools

Five tools were used to collect the data for this study, including (i) the sociodemographic, anthropometric, and clinical tool, (ii) the Hospital Anxiety and Depression Scale (HADS) [12], (iii) Atlanta Heart Failure Knowledge Test, (IV) Self-Care OF Heart Failure Index, and (v) the Short Form-36 Health Survey (SF-36). These data were collected either by administering a questionnaire or by reviewing medical records.

#### 2.7.1. Sociodemographic, Anthropometric, and Clinical Tools

The sociodemographic tool included sociodemographic characteristics and clinical variables. The following sociodemographic variables were collected: age, gender, level of education, body mass index (BMI), marital status, employment status, place of residence, living arrangements, occupation status, monthly income, smoking status, physical activity, and insurance status. The researcher used medical records to collect clinical variables, including physical activity (patient self-reported), NYHA heart failure classification, the presence and number of other comorbid diseases, medication-related information (obtained from medical records), self-measurement-related information, LVEF, history of HTN and DM, and time since HF diagnosis. Smoking status and sleeping difficulty were recorded as self-reported binary variables (yes/no), physical activity was recorded using the participant’s self-classification (active/not active), monthly income was recorded in Jordanian dinars and analyzed continuously, and comorbidity burden was the unweighted count of documented hypertension, diabetes mellitus, coronary artery disease, hypercholesterolemia, and atrial fibrillation (range 0–5).

The EF is an important value used to determine whether the heart muscle is capable of sufficiently pumping blood, and to track and diagnose systolic heart failure. The EF was measured by echocardiography and classified as HF with reduced ejection fraction (HFrEF) when LVEF ≤ 40%, HF with mildly reduced ejection fraction (HFmrEF) when LVEF was 41–49%, and HF with preserved ejection fraction (HFpEF) when LVEF was ≥50% [40]. The NYHA classification comprises four categories (Class I–IV) to classify the impact of HF symptoms on patients’ ability to walk or exercise and the level of limitation during any physical activity [41]. The study tools were translated into Arabic by an experienced bilingual nursing academic, followed by back-translation into English by another experienced academic, who was blinded to the original version.

BMI was recorded as a continuous variable and calculated as body weight (kg) divided by height (m^2^) to estimate body fat percentage. According to the literature, BMI is classified into five categories: underweight (BMI < 18.5), normal weight (BMI 18.5–24.9), overweight (BMI 25–29.9), mild obesity (BMI 30–34.9), and morbid obesity (BMI ≥ 35) [42,43].

#### 2.7.2. Hospital Anxiety and Depression Scale (HADS)

This instrument evaluates anxiety and depression without addressing somatic symptoms. It consists of 14 items, with 7 focused on anxiety and 7 on depression. Participants rate their symptoms on a 0–3 scale, with each subscale having a score range from 0 to 21 [12]. Higher scores reflect greater frequency and severity of symptoms. The scores are categorized as follows: 0–7 (normal), 8–10 (mild), 11–14 (moderate), and 15–21 (severe anxiety/depression) [12]. Previous studies have reported the following psychometric properties for the Arabic version of the HADS: Cronbach’s α for the anxiety and depression subscales were 0.78 and 0.87, respectively [12,44,45,46,47]. The Arabic version of the HADS was utilized in this study [12]. In the present sample, Cronbach’s α was 0.796 for the anxiety subscale and 0.716 for the depression subscale.

#### 2.7.3. The Atlanta Heart Failure Knowledge Test

The Atlanta Heart Failure Knowledge Test (AHFKT) was developed to evaluate individuals’ knowledge of heart failure (HF), its management, and self-care strategies. Originally published in 2009, it is accessible to healthcare providers [48,49]. The revised version, Version 2, contains 30 questions that assess patient education on topics such as the HF disease process, diet and nutrition (including restrictions on sodium and fluids), medications, symptoms, and lifestyle behaviors like daily weight tracking and physical activity [48,49]. The AHFKT has been employed in a variety of research studies [50,51,52,53,54].

The test gives 1 point for each correct answer, with no extra points for any particular question. The total score is the sum of correct answers, and incorrect or skipped questions get 0 points. For analysis, the score can range from 0 to 30 or be converted into a percentage (0% to 100%) for easier comparison. Although some questions have more than two choices, the test uses a simple right/wrong scoring method, as it is meant to measure specific knowledge [49]. The content validity indexes (CVI) for clarity (0.81) and relevance (0.92) are acceptable, and the overall content validity index (S-CVI/Ave) is excellent at 0.96. Furthermore, a factor analysis by Reilly, Higgins, Smith, Gary, Robinson, Clark, McCarty, and Dunbar [49] showed that the test is reliable and consistent, with a Cronbach’s alpha of 0.87.

The AHFKT had not previously been available in Arabic, the official language of Jordan. To adapt it for use in Jordan, we applied the forward and back-translation methods [55,56,57]. A bilingual nursing expert with U.S. advanced degrees first translated the scale from English to Arabic, followed by another translator who back-translated it into English. The research team reviewed both versions for consistency. The Arabic version was then tested with ten heart failure patients (five females and five males) who frequently visited the cardiac clinic. They reported that the tool was clear, understandable, and culturally appropriate. In the present sample, Kuder–Richardson 20 reliability was 0.670. Although this estimate was below the commonly cited 0.70 benchmark, fixed cutoffs should be interpreted cautiously for broad knowledge tests because their items assess related but nonredundant content, and multiple-choice items scored as correct or incorrect may yield lower internal-consistency estimates than open-ended items [58,59].

#### 2.7.4. Self-Care of Heart Failure Index (SCHFI) (v. 7.2)

The revised SCHFI (version 7.2) is a tool designed to evaluate self-care capabilities in individuals with heart failure. It contains 39 questions that cover three primary areas of self-care: self-care maintenance, symptom perception, and self-care management. In addition, it assesses self-care confidence, which is not a part of selfcare, but has a strong impact on it. The SCHFI uses a Likert scale for responses. The self-care maintenance section includes 10 questions that focus on health-related behaviors such as diet, exercise, follow-up care, and infection prevention, with response options ranging from “never” (1) to “always” (5). The symptom perception section consists of 9 items that evaluate the frequency of heart failure symptoms, including breathlessness, swelling, weight gain, and fatigue. It also includes 2 questions that assess how quickly the patient identified these symptoms as related to heart failure, with responses ranging from “not applicable” (no symptoms) or 0 (did not recognize) to 5 (recognized quickly).

The self-care management section contains 8 questions, 7 of which assess the likelihood of the respondent engaging in behaviors commonly used to manage heart failure symptoms, with answers ranging from 1 (unlikely) to 5 (very likely). One question asks about the patient’s confidence in the effectiveness of the most recent treatment they received, with responses ranging from 0 (no action) or 1 (uncertain) to 5 (very confident). The total score is then standardized to a scale from 0 to 100, with higher scores indicating a higher level of self-care [60,61].

Importantly, the SCHFI management scale is conditionally administered and applies only to patients reporting recent symptoms. Therefore, absent management scores reflect structural inapplicability rather than missing data.

The study used the Arabic version of the revised Self-Care of Heart Failure Index (SCHFI V.7-2), which is publicly accessible [62,63]. The Arabic version has demonstrated satisfactory psychometric properties in previous studies [64,65,66]. In the present sample, Cronbach’s α was 0.803 for maintenance, 0.840 for symptom perception (427 complete eligible cases), 0.747 for management (476 applicable participants), and 0.946 for self-efficacy.

#### 2.7.5. Short Form-36 Health Survey (SF-36)

The Arabic SF-36 Version 1 used in this study has previously been validated in northern Jordan and comprises 36 items across eight domains: physical functioning, role limitations due to physical health, bodily pain, general health, vitality, social functioning, role limitations due to emotional problems, and mental health. Consistent with Jordanian and regional applications of the SF-36, item responses were recoded according to the SF-36 Version 1 scoring direction so that 0 represented the poorest and 100 the best health state, and the recoded items were averaged within their respective domains, with higher scores indicating better health [67,68]. For the primary analyses, the unweighted SF-36 physical four-domain composite was calculated as (vitality + social functioning + role limitations due to emotional problems + mental health)/4. This approach retained the original 0–100 domain metric, avoided applying external population weights, and is supported as a simple method for constructing correlated physical and mental summary scores [69]. In the present sample, Cronbach’s α across the four constituent domain scores was 0.707 for the SF-36 physical four-domain composite and 0.717 for the SF-36 mental four-domain composite. As a sensitivity analysis, conventional US norm-based SF-36 Version 1 PCS and MCS T-scores were calculated by standardizing the eight domains using published US means and standard deviations, applying the published factor-score coefficients, and transforming the weighted sums to a mean of 50 and SD of 10 [70]. Because these means, standard deviations, and coefficients were developed in a US population and their applicability to Jordan has not been established, the US norm-based scores were not used as the primary outcomes.

### 2.8. Statistical Analysis

Statistical analysis was conducted using R version 4.5.3. Descriptive statistics were reported as means (SD), medians (IQR), or frequencies (%), as appropriate. Primary outcomes were the SF-36 physical and mental four-domain composites (0–100). Separate ordinary least-squares models were fitted for each outcome using HC3 robust standard errors [71]. The covariate set included age, sex, BMI, NYHA class, EF category, SCHFI maintenance, symptom perception, management, and self-efficacy scores, AHFKT percentage, education, income, work status, residency, marital status, smoking, physical activity, sleeping difficulty, comorbidity count (0–5), HF duration, and prior HF admission. HADS was represented by its anxiety and depression subscale scores entered simultaneously, consistent with the instrument’s two-subscale structure. Predictors were prespecified before outcome modeling and were not selected according to univariable statistical significance. Selection was guided by the adapted Wilson and Cleary HRQoL framework and prior HF literature summarized in the Introduction [26,28,32]. Age and sex represented core demographic characteristics; education, income, work status, residency, and marital status represented socioeconomic resources, healthcare access, and social support; BMI, NYHA class, EF category, comorbidity count, HF duration, and prior HF admission represented anthropometric status, functional limitation, cardiac phenotype, and cumulative clinical burden; smoking, physical activity, and sleeping difficulty represented behavioral and symptom-related factors; HADS anxiety and depression represented psychological distress; and the SCHFI domains and AHFKT represented self-care behaviors, symptom appraisal, self-efficacy, and HF knowledge. The same prespecified covariate set was retained in both outcome models to facilitate comparison and avoid data-driven variable selection. Categorical predictors used treatment coding with the following reference groups: male sex, NYHA class I, HFpEF (≥50%), no formal education, working, urban residence, married, nonsmoker, active, no sleeping difficulty, HF diagnosed at the current visit, and no prior HF admission. Both unstandardized coefficients (B) and standardized coefficients (β) were reported.

Missingness was evaluated for every model variable. All 516 participants had computable SF-36, HADS anxiety and depression, AHFKT, and SCHFI maintenance, symptom perception, and self-efficacy scores. SCHFI management was structurally inapplicable for 40 participants who had no relevant symptom-treatment episode; listwise deletion therefore yielded 476 observations in both adjusted models, with no additional exclusions. Medication variables were not entered because 232 participants (45.0%) could not identify their HF medications.

For EF-category comparisons, unadjusted HC3 omnibus tests, Tukey-adjusted pairwise comparisons with simultaneous 95% confidence intervals, Hedges’ g, and omega-squared effect sizes were calculated. Adjusted EF omnibus tests, covariate-standardized marginal means with 95% confidence intervals, and Tukey-adjusted contrasts were obtained from the primary models. Linearity was assessed using residual plots, RESET and Rainbow tests, and quadratic-term tests for continuous predictors with false-discovery-rate adjustment. Residual distribution, heteroscedasticity, multicollinearity, and influence were evaluated using Q–Q plots, residual skewness and kurtosis, the Breusch–Pagan test, GVIF, studentized residuals, Cook’s distance, and leverage. Because the outcomes were bounded and residual normality tests were statistically significant, median regression with 2000 xy-pair bootstrap replications was used as a sensitivity analysis [72]. However, residual skewness and excess kurtosis were modest, indicating only minor departures from exact normality rather than serious model misspecification. Therefore, OLS with HC3 robust standard errors was retained as the primary analysis. Additional sensitivity models excluded NYHA class from the physical model, excluded both HADS subscales from the mental model, and used US norm-based SF-36 Version 1 PCS and MCS scores. Complete diagnostic and sensitivity results are provided in Supplementary File S1.

## 3. Results

The study sample included 516 participants. All participants had computable SF-36, HADS anxiety and depression, AHFKT, and SCHFI maintenance, symptom perception, and self-efficacy scores. Management scale scores from the SCHFI were unavailable for 40 participants because the scale is only administered to individuals who reported recent symptoms; thus, these data are structurally inapplicable rather than missing. Participant sociodemographic characteristics are reported in Table 1. The median age was 60 years (IQR 53–64.5); 81% were male, 61% resided in urban areas, and 93% were married.

The clinical characteristics of the sample are presented in Table 2. Participants had a median BMI of 28.26 kg/m^2^ (IQR = 25.45–32.14). Most participants reported functional limitations, with 50% classified as NYHA class II and 30% as class III. Regarding ejection fraction (EF), 58% had heart failure with reduced ejection fraction (HFrEF; EF ≤ 40%), whereas only 9.1% had preserved ejection fraction (HFpEF; EF ≥ 50%).

Current smoking was reported by 43% of participants, 50% classified themselves as physically inactive, and 46% reported sleeping difficulty. Hypertension (53%), coronary artery disease (48%), and diabetes mellitus (42%) were common. Among the 284 participants able to identify their medications, loop diuretics were reported by 64.8%, vasodilators by 44.0%, beta blockers by 35.6%, and valsartan by 31.7%; 232 participants (45.0% of the full sample) could not identify their HF medications.

Unadjusted and adjusted SF-36 composite scores by EF category are summarized in Table 3. Before adjustment, both SF-36 physical and mental four-domain composite scores differed across EF categories (physical *p* < 0.001, ω^2^ = 0.044; mental *p* < 0.001, ω^2^ = 0.026).

For the SF-36 physical four-domain composite, all three unadjusted pairwise differences remained significant after Tukey correction: HFmrEF versus HFpEF, mean difference −10.96 (simultaneous 95% CI −18.89 to −3.03; *p* = 0.004; Hedges’ g = −0.52); HFrEF versus HFpEF, −15.79 (−23.27 to −8.31; *p* < 0.001; g = −0.77); and HFrEF versus HFmrEF, −4.83 (−9.57 to −0.09; *p* = 0.045; g = −0.23).

For the SF-36 mental four-domain composite, only HFrEF versus HFpEF remained significant after Tukey correction (mean difference −11.56, simultaneous 95% CI −18.87 to −4.25; *p* < 0.001; g = −0.57). HFmrEF versus HFpEF (*p* = 0.082) and HFrEF versus HFmrEF (*p* = 0.058) were not significant. After covariate adjustment, the EF omnibus test was not significant for either outcome, and none of the adjusted pairwise contrasts remained significant after Tukey correction.

Questionnaire outcomes are summarized in Table 4. Median HADS anxiety and depression scores were both 8. The median AHFKT score was 53%, and the highest SCHFI median was observed for self-efficacy (68). The median SF-36 physical and mental four-domain composite scores were 46.25 and 49.63, respectively.

The expanded SF-36 physical four-domain composite model included 476 participants and explained 58.5% of the outcome variance (adjusted R^2^ = 0.558; Table 5). NYHA class II (B = −9.44, 95% CI −13.19 to −5.70; *p* < 0.001) and class III (B = −14.63, 95% CI −18.84 to −10.42; *p* < 0.001) were associated with lower SF-36 physical four-domain composite scores. The adjusted EF omnibus test was not significant, where F(2, 446) = 2.21 and *p* = 0.111. In sensitivity analyses using median regression, EF category was significantly associated with physical HRQoL; however, this association was not observed in the primary OLS model. Although the HFmrEF and HFrEF reference-category coefficients had nominal *p*-values of 0.041 and 0.050, neither contrast remained significant after Tukey adjustment (*p* = 0.101 and *p* = 0.123, respectively).

Physical inactivity (B = −4.92; *p* = 0.002), sleeping difficulty (B = −4.08; *p* = 0.013), retirement (B = −4.37; *p* = 0.012), higher HADS anxiety (B = −0.90 per point; standardized β = −0.19; *p* < 0.001), and higher HADS depression (B = −1.40 per point; standardized β = −0.28; *p* < 0.001) were independently associated with lower physical scores. Higher SCHFI self-efficacy was associated with higher physical scores (B = 0.08 per point; *p* = 0.046).

BMI approached but did not reach statistical significance (*p* = 0.051). Age, sex, education, income, residency, marital status, smoking, AHFKT, the other SCHFI subscales, comorbidity count, HF duration, and prior HF admission were not independently associated with the SF-36 physical four-domain composite (all *p* > 0.05).

The expanded SF-36 mental four-domain composite model included 476 participants and explained 56.0% of the outcome variance (adjusted R^2^ = 0.532; Table 6). NYHA class II (B = −6.55, 95% CI −10.43 to −2.67; *p* < 0.001), NYHA class III (B = −8.77, 95% CI −13.01 to −4.52; *p* < 0.001), physical inactivity (B = −5.51; *p* < 0.001), higher HADS anxiety (B = −1.68 per point; standardized β = −0.36; *p* < 0.001), and higher HADS depression (B = −1.11 per point; standardized β = −0.22; *p* < 0.001) were independently associated with lower SF-36 mental four-domain composite scores. Higher SCHFI self-efficacy was associated with higher mental scores (B = 0.11 per point; *p* = 0.010).

The EF omnibus test was not significant, where F(2, 446) = 0.49 and *p* = 0.613, and neither HFmrEF nor HFrEF differed from HFpEF after Tukey adjustment. Sleeping difficulty was not significant (*p* = 0.127). The remaining sociodemographic, clinical, knowledge, and self-care covariates were also not independently associated with the SF-36 mental four-domain composite (all *p* > 0.05).

The block containing comorbidity count, HF duration, and prior HF admission was not significant for either the physical (*p* = 0.497) or mental (*p* = 0.500) model.

Sensitivity analyses are reported in Supplementary File S1. Models excluding NYHA from the physical model and both HADS subscales from the mental model clarified the expected construct overlap without reversing the main conclusions. Median regression estimates for anxiety and depression remained negative and statistically significant for both outcomes; however, the EF omnibus test was significant in the physical median model (*p* = 0.023) but not in the primary OLS model. No observation had Cook’s distance > 1, and influence-exclusion refits did not alter the main interpretation. US norm-based SF-36 Version 1 PCS and MCS models yielded R^2^ values of 0.464 and 0.453, respectively, and some differences in individual coefficients, supporting their use as sensitivity analyses rather than primary outcomes.

## 4. Discussion

This study provides insight into HRQoL and the factors associated with it among individuals with HF. Patients’ reported health was considered in relation to cardiac, functional, psychosocial, behavioral, and self-care efficacy measures. Physical and mental HRQoL were analyzed as distinct composite outcomes.

The sociodemographic and clinical findings for the study participants correspond to HF patients reported within regional and global registry data, specifically regarding the large proportion of older males with multiple cardiometabolic comorbidities [40,73]. The high prevalence of hypertension, diabetes mellitus, coronary artery disease, and overweight illustrates the clinical complexity of this HF population. Comprehensive clinical assessment remains important, although comorbidity count was not independently associated with either composite outcome after adjustment.

SF-36 physical and mental four-domain composite scores differed across EF categories, but EF category was not associated with either composite in the primary adjusted omnibus tests. The attenuation of these differences after covariate adjustment supports the view that EF alone does not characterize patient-reported health [74]. Accordingly, cardiac performance indices should be considered alongside patient-reported measures rather than used as surrogates for well-being.

NYHA functional class was the most consistent clinical factor associated with both physical and mental HRQoL. Recent research suggests the existence of a positive relationship between higher levels of the NYHA classification system and reduced performance in all areas of HRQoL, reflecting a considerable impairment of physical function, mental well-being, and participation in patients classified as belonging to the NYHA III and IV levels, in comparison with patients belonging to lower classification levels. This finding is consistent with contemporary research identifying NYHA class as a major correlate of health-related QoL in patients with HF [31,35,75,76].

The main integrating factor of the NYHA classification system refers to the existing confluence of the patient’s burden of symptoms, performance during exercise, and ability to perform everyday activity, well representing the patient’s reality in HF, as evident in contemporary research findings on HF patients diagnosed through the application of this system of classification [75,76].

Anxiety and depressive symptoms were independently associated with lower HRQoL. Higher anxiety and depression scores were associated with poorer physical and mental functioning across HF severity levels [12,77,78]. This finding is consistent with the meta-analysis that showed psychological morbidity to be common in patients with heart failure and that these variables are associated with lower quality of life, increased hospitalization, and increased all-cause mortality [79]. Both subscales were independently associated with both composite outcomes in the primary models. Depression had the larger standardized association with the SF-36 physical four-domain composite, whereas anxiety had the larger standardized association with the SF-36 mental four-domain composite; these differences are descriptive because the coefficients were not formally compared. Overlap between the HADS subscales and SF-36 mental health content may contribute to the strength of these associations.

Behavioral variables were associated with HRQoL in patients with HF, specifically in terms of inactivity and sleep disturbances. Physical inactivity was independently associated with lower physical and SF-36 mental four-domain composite scores, confirming the existing literature suggesting that regular physical activity improves tolerance to exercise, decreases depressive symptoms, and improves HRQoL in patients with HF [80,81,82]. Sleeping difficulty, reported by almost half of the participants, was associated with lower physical HRQoL, as the existing literature suggests that irregular sleep patterns contribute to increased fatigue, decreased functional capacity, and decreased daytime productivity in patients with HF [83,84].

After adjustment, BMI did not reach statistical significance for the SF-36 physical four-domain composite (*p* = 0.051); therefore, no independent association was inferred. Among the self-care areas, self-care efficacy was independently associated with higher physical and SF-36 mental four-domain composite scores. This aligns with the theory of self-care in HF, which suggests that confidence is a factor in translating knowledge and skills into self-care practice [85,86]. AHFKT knowledge was not independently associated with either composite after adjustment. These findings support further evaluation of strategies intended to strengthen patients’ self-care confidence. Socioeconomic and occupational variables were also evaluated. Retirement was associated with a lower SF-36 physical four-domain composite but not with the SF-36 mental four-domain composite, which is in line with evidence that suggests a link between socioeconomic variables and care processes as well as treatment and supportive environments [87,88].

Such findings highlight the significance of addressing both social and economic issues in designing patient-centric interventions for HF. Taken together, the findings of this study are consistent with the conceptual understanding of HF as a “biopsychosocial phenomenon” in which HF illness stage, distress, personality style, and self-efficacy may be associated with QOL in HF patients. This could be significant in resource-limited healthcare settings, where improving QOL may be as important as improving survival.

This multicenter study provides a comprehensive understanding of health-related quality of life (HRQoL) among patients with heart failure (HF) in Jordan across different ejection fraction categories. The findings indicate that HRQoL was associated with functional limitations, anxiety and depressive symptoms, and self-care confidence in addition to cardiac measures. The physical and mental composites were interpreted separately; their numerical difference was not treated as evidence of disproportionate physical impairment.

Notably, ejection fraction alone did not sufficiently explain variations in HRQoL, reinforcing the limitations of relying solely on traditional cardiac indicators to evaluate patient outcomes. This finding supports a shift toward a more holistic, patient-centered approach to HF management—one that prioritizes functional recovery, psychological well-being, and self-efficacy alongside optimal medical therapy. Such an approach is particularly relevant in diverse clinical settings, where improving HRQoL may represent a primary goal of care regardless of ejection fraction status.

This study has important implications for clinical practice, research, and community health. The findings indicate that effective heart failure care should extend beyond ejection fraction, as this parameter alone does not adequately capture patients’ lived experiences. Incorporating routine assessments of health-related quality of life, functional status, anxiety and depressive symptoms, and self-care confidence into clinical practice may enable clinicians to better identify patient needs and tailor care accordingly. Patient-centered, multidisciplinary approaches that address physical functioning, mental well-being, sleep quality, and self-management skills may therefore inform efforts to improve outcomes that matter most to patients, alongside traditional clinical measures.

From a research, community, and health system perspective, these findings underscore the importance of community-based and nurse-led interventions to enhance functional capacity and self-efficacy, particularly among older adults and socioeconomically vulnerable populations. The systematic use of patient-reported outcome measures may support more responsive, equitable, and patient-informed care delivery, as well as guide service planning and evaluation. Future longitudinal and interventional research is needed to clarify causal pathways and to evaluate culturally appropriate, community-focused strategies designed to improve health-related quality of life across the full spectrum of heart failure, irrespective of ejection fraction.

This study has several limitations. Its cross-sectional design precludes causal inference, and the association models include conceptually overlapping constructs: NYHA class reflects activity limitation measured by the SF-36 physical four-domain composite, while the HADS anxiety and depression subscales overlap with SF-36 mental health content. Recruitment from outpatient clinics in northern Jordan and exclusion of NYHA class IV and severe comorbid conditions may limit generalizability. The HFpEF subgroup was small (47 participants in the full sample and 40 in the complete-case models), reducing precision. Physical activity, sleeping difficulty, medication identification, and self-care were self-reported; physical activity and sleeping difficulty were recorded using broad categories without documented operational thresholds. Forty participants had structurally inapplicable SCHFI management scores, reducing the adjusted sample to 476. Medication use was not modeled because 45.0% of participants could not identify their medications. The same Arabic SF-36 Version 1 instrument and 0–100 domain scoring used in this study have previously been validated and applied in Jordan and the region [61,62]. The limitation concerns only the conventional norm-based PCS and MCS T-score algorithm, which additionally depends on US-derived domain means, standard deviations, and factor-score coefficients [64]. Because these parameters were not developed for the Jordanian population and their applicability in Jordan has not been established, the primary analyses retained the 0–100 domain metric and used unweighted physical and mental composites; US norm-based PCS and MCS scores were examined only in sensitivity analyses.

Although participants were recruited from two outpatient clinics, both hospitals are located in the same geographic area and serve a similar catchment population with comparable sociodemographic, cultural, linguistic, and clinical characteristics. Therefore, the hospital site was unlikely to have substantially influenced the findings. However, recruitment from only two healthcare settings may limit the generalizability of the results, which has been acknowledged as a study limitation.

## 5. Conclusions

This multicenter study describes physical and mental HRQoL among Jordanian adults with HFrEF, HFmrEF, and HFpEF. NYHA functional class, anxiety and depressive symptoms, physical inactivity, and self-care efficacy were the most consistent factors associated with the SF-36 composites, whereas EF category was not significant in the adjusted omnibus tests. These cross-sectional associations support patient-centered assessment of functional status, psychological symptoms, health behaviors, and self-care alongside cardiac measures, but they do not establish causal effects.

## Figures and Tables

**Table 1:** Demographic characteristics of the study participants (N = 516).

Variable		Count (%)
Age (years)		60 (53–64.5)
Sex	Male	418 (81.0)
	Female	98 (19.0)
Residency	Urban	316 (61.0)
	Rural	200 (39.0)
Education	Higher	226 (44.0)
	No education	26 (5.0)
	Elementary	59 (11.0)
	Secondary	205 (40.0)
Work status	Working	158 (31.0)
	Retired	212 (41.0)
	Not working	146 (28.0)
Income		400 (200–500)
Marital status	Married	482 (93.0)
	Unmarried	34 (6.6)

Values are reported as Median (Q1, Q3); n (%).

**Table 2:** Clinical profile and medications.

Variable		Count (%)
BMI		28.26 (25.45, 32.14)
NYHA class	I	104 (20.0)
	II	258 (50.0)
	III	154 (30.0)
EF	HFpEF (≥50%)	47 (9.1)
	HFmrEF (41–49%)	168 (33.0)
	HFrEF (≤40%)	301 (58.0)
Current smoker		220 (43.0)
Physical activity level	Active	258 (50.0)
	Not active	258 (50.0)
Sleeping difficulty		235 (46.0)
Medical history	HTN	272 (53.0)
	DM	215 (42.0)
	CAD	250 (48.0)
	Hypercholesteremia	166 (32.0)
	AF	63 (12.0)
HF medications	Aldosterone antagonist	50/284 (17.6)
	Digoxin	50/284 (17.6)
	ACEI/ARB	51/284 (18.0)
	Beta blocker	101/284 (35.6)
	Loop diuretic	184/284 (64.8)
	Vasodilator	125/284 (44.0)
	Calcium-channel blocker	33/284 (11.6)
	Ivabradine	24/284 (8.5)
	Valsartan	90/284 (31.7)
Medication identification	Could not identify HF medications	232/516 (45.0)

Median (Q1, Q3); n (%). Medication percentages use N = 284 participants who could identify their HF medications; the numerator and denominator are displayed. Abbreviations: BMI, body mass index; NYHA, New York Heart Association; HTN, hypertension; DM, diabetes mellitus; CAD, coronary artery disease; AF, atrial fibrillation; ACEI/ARB, angiotensin-converting enzyme inhibitor/angiotensin receptor blocker; CCB, calcium-channel blocker; EF, ejection fraction; HFrEF, HF with reduced EF; HFmrEF, HF with mildly reduced EF; HFpEF, HF with preserved EF.

**Table 3:** Unadjusted and adjusted SF-36 physical and mental four-domain composite scores by ejection fraction category.

Outcome/Statistic	Total Sample	HFpEF (≥50%)	HFmrEF (41–49%)	HFrEF (≤40%)	EF Omnibus *p*	Effect Size
SF-36 physical four-domain composite, unadjusted mean (SD)	48.80 (21.14)	61.58 (20.03)	50.62 (21.09)	45.79 (20.52)	<0.001	ω^2^ = 0.044
SF-36 physical four-domain composite, adjusted mean (95% CI)	—	53.12 (47.99–58.25)	47.17 (44.62–49.73)	47.66 (46.01–49.32)	0.111	—
SF-36 mental four-domain composite, unadjusted mean (SD)	51.88 (20.57)	60.92 (19.54)	53.87 (20.40)	49.36 (20.36)	<0.001	ω^2^ = 0.026
SF-36 mental four-domain composite, adjusted mean (95% CI)	—	53.57 (48.54–58.59)	51.76 (49.43–54.09)	50.99 (49.26–52.72)	0.613	—

Unadjusted rows use the full HRQoL sample (N = 516); adjusted marginal means use the complete-case model sample (n = 476) and HC3 covariance. Pairwise *p*-values and simultaneous confidence intervals were Tukey-adjusted. Higher scores indicate better HRQoL. The nonstandard mean of all eight SF-36 domains was removed.

**Table 4:** Measurement scores and SF-36 composite outcomes (N = 516).

Measure	Scale	Median (IQR)
HADS	Anxiety	8 (4–11)
	Depression	8 (5–11)
AHFKT	Knowledge, %	53 (43–60)
SCHFI	Maintenance	50 (35–65)
	Symptom perception	43 (30–61)
	Management	55 (39–70)
	Self-efficacy	68 (50–86)
SF-36	Physical four-domain composite	46.25 (32.97–63.28)
	Mental four-domain composite	49.63 (36.00–68.39)

Values are median (IQR). SCHFI management was applicable to 476 participants. Abbreviations: HADS, Hospital Anxiety and Depression Scale; AHFKT, Atlanta Heart Failure Knowledge Test; SCHFI, Self-Care of Heart Failure Index; SF-36, short form 36-Item Health Survey Version 1.0.

**Table 5:** Expanded OLS model for the SF-36 physical four-domain composite (n = 476).

Characteristic	Category	B	95% CI	Standardized β	*p*-Value
Age, years		−0.03	−0.19 to 0.13	−0.01	0.703
Sex	Male (reference)	—	—	—	
	Female	−1.28	−5.56 to 3.00	−0.02	0.557
BMI, kg/m^2^		−0.22	−0.43 to 0.00	−0.06	0.051
NYHA class	I (reference)	—	—	—	
	II	−9.44	−13.19 to −5.70	−0.23	<0.001
	III	−14.63	−18.84 to −10.42	−0.33	<0.001
EF category	HFpEF (≥50%; reference)	—	—	—	
	HFmrEF (41–49%)	−5.94	−11.63 to −0.26	−0.13	0.041
	HFrEF (≤40%)	−5.45	−10.91 to 0.01	−0.13	0.050
SCHFI	Maintenance	0.01	−0.07 to 0.10	0.01	0.740
	Symptom perception	−0.08	−0.17 to 0.01	−0.08	0.068
	Management	0.07	−0.03 to 0.17	0.07	0.192
	Self-efficacy	0.08	0.00 to 0.17	0.10	0.046
AHFKT, %		−0.07	−0.18 to 0.04	−0.04	0.217
Education	No formal education (reference)	—	—	—	
	Elementary	−0.54	−8.35 to 7.26	−0.01	0.891
	Secondary	1.45	−5.29 to 8.19	0.03	0.673
	Higher	2.22	−4.88 to 9.32	0.05	0.540
Monthly income, JOD		0.00	−0.00 to 0.01	0.07	0.130
Work status	Working (reference)	—	—	—	
	Retired	−4.37	−7.76 to −0.97	−0.10	0.012
	Not working	−1.79	−6.09 to 2.52	−0.04	0.416
Residency	Urban (reference)	—	—	—	
	Rural	−0.76	−3.59 to 2.06	−0.02	0.596
Marital status	Married (reference)	—	—	—	
	Unmarried	2.77	−2.47 to 8.02	0.03	0.299
Current smoker	No (reference)	—	—	—	
	Yes	−1.09	−4.07 to 1.89	−0.03	0.473
Physical activity	Active (reference)	—	—	—	
	Not active	−4.92	−8.07 to −1.77	−0.12	0.002
Sleeping difficulty	No (reference)	—	—	—	
	Yes	−4.08	−7.28 to −0.87	−0.10	0.013
HADS anxiety		−0.90	−1.31 to −0.49	−0.19	<0.001
HADS depression		−1.40	−1.87 to −0.94	−0.28	<0.001
Comorbidity count	0–5	0.06	−0.98 to 1.10	0.00	0.913
HF duration	At current visit (reference)	—	—	—	
	<1 year	1.79	−2.77 to 6.36	0.04	0.441
	>1 year	3.36	−0.60 to 7.31	0.08	0.096
Prior HF admission	No (reference)	—	—	—	
	Yes	−1.88	−4.89 to 1.13	−0.05	0.220
R^2^		0.585			
Adjusted R^2^		0.558			
No. observations		476			

B denotes the unstandardized regression coefficient; β denotes the standardized coefficient. Confidence intervals and *p*-values use HC3 robust standard errors. The EF omnibus test was F(2, 446) = 2.21, *p* = 0.111. Abbreviations: CI, confidence interval; HADS, Hospital Anxiety and Depression Scale; AHFKT, Atlanta Heart Failure Knowledge Test; SCHFI, Self-Care of Heart Failure Index; HF, heart failure; NYHA, New York Heart Association.

**Table 6:** Expanded OLS model for the unweighted SF-36 mental four-domain composite (n = 476).

Characteristic	Category	B	95% CI	Standardized β	*p*-Value
Age, years		0.03	−0.13 to 0.20	0.02	0.688
Sex	Male (reference)	—	—	—	
	Female	−0.97	−5.21 to 3.27	−0.02	0.653
BMI, kg/m^2^		0.08	−0.15 to 0.30	0.02	0.504
NYHA class	I (reference)	—	—	—	
	II	−6.55	−10.43 to −2.67	−0.16	<0.001
	III	−8.77	−13.01 to −4.52	−0.20	<0.001
EF category	HFpEF (≥50%; reference)	—	—	—	
	HFmrEF (41–49%)	−1.81	−7.33 to 3.72	−0.04	0.521
	HFrEF (≤40%)	−2.58	−7.96 to 2.80	−0.06	0.347
SCHFI	Maintenance	0.01	−0.08 to 0.09	0.01	0.890
	Symptom perception	−0.05	−0.14 to 0.04	−0.05	0.313
	Management	−0.01	−0.10 to 0.08	−0.01	0.804
	Self-efficacy	0.11	0.03 to 0.19	0.13	0.010
AHFKT, %		0.04	−0.07 to 0.15	0.03	0.444
Education	No formal education (reference)	—	—	—	
	Elementary	0.40	−7.32 to 8.12	0.01	0.920
	Secondary	4.94	−1.63 to 11.51	0.12	0.140
	Higher	1.64	−5.36 to 8.63	0.04	0.645
Monthly income, JOD		0.00	−0.00 to 0.01	0.05	0.229
Work status	Working (reference)	—	—	—	
	Retired	−2.23	−5.61 to 1.14	−0.05	0.193
	Not working	−1.54	−5.63 to 2.55	−0.03	0.459
Residency	Urban (reference)	—	—	—	
	Rural	0.05	−2.72 to 2.82	0.00	0.971
Marital status	Married (reference)	—	—	—	
	Unmarried	0.96	−4.92 to 6.85	0.01	0.748
Current smoker	No (reference)	—	—	—	
	Yes	−0.46	−3.42 to 2.50	−0.01	0.761
Physical activity	Active (reference)	—	—	—	
	Not active	−5.51	−8.72 to −2.29	−0.14	<0.001
Sleeping difficulty	No (reference)	—	—	—	
	Yes	−2.40	−5.49 to 0.69	−0.06	0.127
HADS anxiety		−1.68	−2.11 to −1.25	−0.36	<0.001
HADS depression		−1.11	−1.60 to −0.61	−0.22	<0.001
Comorbidity count	0–5	0.42	−0.64 to 1.47	0.03	0.438
HF duration	At current visit (reference)	—	—	—	
	<1 year	−3.25	−7.59 to 1.10	−0.07	0.143
	>1 year	−1.01	−4.75 to 2.74	−0.02	0.597
Prior HF admission	No (reference)	—	—	—	
	Yes	−0.80	−3.94 to 2.34	−0.02	0.618
R^2^		0.560			
Adjusted R^2^		0.532			
No. observations		476			

B denotes the unstandardized regression coefficient; β denotes the standardized coefficient. Confidence intervals and *p*-values use HC3 robust standard errors. The EF omnibus test was F(2, 446) = 0.49, *p* = 0.613. Abbreviations: CI, confidence interval; HADS, Hospital Anxiety and Depression Scale; AHFKT, Atlanta Heart Failure Knowledge Test; SCHFI, Self-Care of Heart Failure Index; HF, heart failure; NYHA, New York Heart Association.

## Data Availability

In accordance with the requirements of our Institutional Review Board and the informed consent provided by participants, the data cannot be made publicly available. However, the data supporting the findings of this study are available from the corresponding author upon reasonable request. Requests will be considered in accordance with ethical approval requirements and applicable institutional policies.

## Supplementary Materials

The following supporting information can be downloaded at: https://www.mdpi.com/article/10.3390/healthcare14162447/s1.

## Abbreviations

AHFKT	Atlanta Heart Failure Knowledge Test
BMI	Body Mass Index
COPD	Chronic Obstructive Pulmonary Disease
DM	Diabetes Mellitus
EF	Ejection Fraction
GVIF	Generalized Variance Inflation Factor
HADS	Hospital Anxiety and Depression Scale
HF	Heart Failure
HRQoL	Health-Related Quality of Life
IRB	Institutional Review Board
LVEF	Left Ventricular Ejection Fraction
MCS	Mental Component Summary (SF-36)
NYHA	New York Heart Association functional classification
PCS	Physical Component Summary (SF-36)
SCHFI	Self-Care of Heart Failure Index
SF-36	Short Form-36 Health Survey
HTN	Hypertension
