# Supplementary material for: Health-Related Quality of Life and Associated Factors in Patients with Heart Failure Across Three Ejection Fraction Categories: A Two-Center Cross-Sectional Study
Source: Healthcare (Basel). 2026 Aug 7;14(16):2447. doi: 10.3390/healthcare14162447 (PMC13511906; doi:10.3390/healthcare14162447)
Supplement: Supplementary file 1 [file healthcare-14-02447-s001.zip › healthcare-4423039-supplementary.pdf]

Supplementary File S1

Model diagnostics and sensitivity analyses for the SF-36 physical and mental four-domain composite models

This supplement reports model diagnostics and sensitivity analyses for primary ordinary least-squares models in which HADS anxiety and depression subscale scores were entered simultaneously. The primary outcomes were unweighted SF-36 physical and mental four-domain composites scored from 0 to 100. Both models used n = 476 complete cases and HC3 robust inference.

S1. Analytic sample and missingness

Table S1

Missingness for variables used in the primary analysis

Variable	Total N	Nonmissing	Missing	Missing, %	Note
Age	516	516	0	0.0	
Sex	516	516	0	0.0	
BMI	516	516	0	0.0	
NYHA class	516	516	0	0.0	
EF category	516	516	0	0.0	
Education	516	516	0	0.0	
Income	516	516	0	0.0	
Work status	516	516	0	0.0	
Residency	516	516	0	0.0	
Marital status	516	516	0	0.0	
Smoking	516	516	0	0.0	
Physical activity	516	516	0	0.0	
Sleeping difficulty	516	516	0	0.0	
HADS anxiety	516	516	0	0.0	
HADS depression	516	516	0	0.0	
AHFKT	516	516	0	0.0	
SCHFI maintenance	516	516	0	0.0	
SCHFI symptom perception	516	516	0	0.0	
SCHFI management	516	476	40	7.8	Structurally not applicable for 40 participants
SCHFI self-efficacy	516	516	0	0.0	
Comorbidity count	516	516	0	0.0	
HF duration	516	516	0	0.0	
Prior HF admission	516	516	0	0.0	
Physical composite	516	516	0	0.0	
Mental composite	516	516	0	0.0	

Note. The 40 SCHFI management values were structurally inapplicable. All other variables in the primary models were complete.

S2. Primary-model results

Table S2.1

Primary-model fit, EF omnibus tests, and clinical-history covariate-block tests

Outcome	n	Residual df	R^2	Adjusted R^2	EF F (df)	EF p	Clinical-history χ^2 (df)	Block p
Physical four-domain composite	476	446	0.585	0.558	2.21 (2, 446)	.111	3.37 (4)	.497
Mental four-domain composite	476	446	0.560	0.532	0.49 (2, 446)	.613	3.35 (4)	.500

Note. The clinical-history block jointly tests comorbidity count, HF duration, and prior HF admission. EF tests use robust F inference; block tests use HC3 Wald χ^2 inference.

Table S2.2

Expanded OLS model for the unweighted SF-36 physical four-domain composite (n = 476)

Characteristic	Category	B	95% CI	Standardized β	p
Age, years		−0.03	−0.19 to 0.13	−0.01	.703
Sex	Male (reference)	—	—	—	
Sex	Female	−1.28	−5.56 to 3.00	−0.02	.557
BMI, kg/m²		−0.22	−0.43 to 0.00	−0.06	.051
NYHA class	I (reference)	—	—	—	
NYHA class	II	−9.44	−13.19 to −5.70	−0.23	< .001
NYHA class	III	−14.63	−18.84 to −10.42	−0.33	< .001
EF category	HFpEF (≥50%; reference)	—	—	—	
EF category	HFmrEF (41–49%)	−5.94	−11.63 to −0.26	−0.13	.041
EF category	HFrEF (≤40%)	−5.45	−10.91 to 0.01	−0.13	.050
SCHFI	Maintenance	0.01	−0.07 to 0.10	0.01	.740
SCHFI	Symptom perception	−0.08	−0.17 to 0.01	−0.08	.068
SCHFI	Management	0.07	−0.03 to 0.17	0.07	.192
SCHFI	Self-efficacy	0.08	0.00 to 0.17	0.10	.046
AHFKT, %		−0.07	−0.18 to 0.04	−0.04	.217
Education	No formal education (reference)	—	—	—	
Education	Elementary	−0.54	−8.35 to 7.26	−0.01	.891
Education	Secondary	1.45	−5.29 to 8.19	0.03	.673
Education	Higher	2.22	−4.88 to 9.32	0.05	.540
Monthly income, JOD		0.00	−0.00 to 0.01	0.07	.130
Work status	Working (reference)	—	—	—	
Work status	Retired	−4.37	−7.76 to −0.97	−0.10	.012
Work status	Not working	−1.79	−6.09 to 2.52	−0.04	.416
Residency	Urban (reference)	—	—	—	
Residency	Rural	−0.76	−3.59 to 2.06	−0.02	.596
Marital status	Married (reference)	—	—	—	
Marital status	Unmarried	2.77	−2.47 to 8.02	0.03	.299
Current smoker	No (reference)	—	—	—	
Current smoker	Yes	−1.09	−4.07 to 1.89	−0.03	.473
Physical activity	Active (reference)	—	—	—	
Physical activity	Not active	−4.92	−8.07 to −1.77	−0.12	.002
Sleeping difficulty	No (reference)	—	—	—	
Sleeping difficulty	Yes	−4.08	−7.28 to −0.87	−0.10	.013
HADS anxiety		−0.90	−1.31 to −0.49	−0.19	< .001
HADS depression		−1.40	−1.87 to −0.94	−0.28	< .001
Comorbidity count	0–5	0.06	−0.98 to 1.10	0.00	.913
HF duration	At current visit (reference)	—	—	—	
HF duration	<1 year	1.79	−2.77 to 6.36	0.04	.441
HF duration	>1 year	3.36	−0.60 to 7.31	0.08	.096
Prior HF admission	No (reference)	—	—	—	
Prior HF admission	Yes	−1.88	−4.89 to 1.13	−0.05	.220
R^2		0.585			
Adjusted R^2		0.558			
No. observations		476			

Note. B is unstandardized; confidence intervals and p values use HC3 covariance. Standardized β = B × SD(Xj)/SD(Y), including dummy columns. Reference categories are shown. HADS anxiety and depression were entered simultaneously.

Table S2.3

Expanded OLS model for the unweighted SF-36 mental four-domain composite (n = 476)

Characteristic	Category	B	95% CI	Standardized β	p
Age, years		0.03	−0.13 to 0.20	0.02	.688
Sex	Male (reference)	—	—	—	
Sex	Female	−0.97	−5.21 to 3.27	−0.02	.653
BMI, kg/m²		0.08	−0.15 to 0.30	0.02	.504
NYHA class	I (reference)	—	—	—	
NYHA class	II	−6.55	−10.43 to −2.67	−0.16	< .001
NYHA class	III	−8.77	−13.01 to −4.52	−0.20	< .001
EF category	HFpEF (≥50%; reference)	—	—	—	
EF category	HFmrEF (41–49%)	−1.81	−7.33 to 3.72	−0.04	.521
EF category	HFrEF (≤40%)	−2.58	−7.96 to 2.80	−0.06	.347
SCHFI	Maintenance	0.01	−0.08 to 0.09	0.01	.890
SCHFI	Symptom perception	−0.05	−0.14 to 0.04	−0.05	.313
SCHFI	Management	−0.01	−0.10 to 0.08	−0.01	.804
SCHFI	Self-efficacy	0.11	0.03 to 0.19	0.13	.010
AHFKT, %		0.04	−0.07 to 0.15	0.03	.444
Education	No formal education (reference)	—	—	—	
Education	Elementary	0.40	−7.32 to 8.12	0.01	.920
Education	Secondary	4.94	−1.63 to 11.51	0.12	.140
Education	Higher	1.64	−5.36 to 8.63	0.04	.645
Monthly income, JOD		0.00	−0.00 to 0.01	0.05	.229
Work status	Working (reference)	—	—	—	
Work status	Retired	−2.23	−5.61 to 1.14	−0.05	.193
Work status	Not working	−1.54	−5.63 to 2.55	−0.03	.459
Residency	Urban (reference)	—	—	—	
Residency	Rural	0.05	−2.72 to 2.82	0.00	.971
Marital status	Married (reference)	—	—	—	
Marital status	Unmarried	0.96	−4.92 to 6.85	0.01	.748
Current smoker	No (reference)	—	—	—	
Current smoker	Yes	−0.46	−3.42 to 2.50	−0.01	.761
Physical activity	Active (reference)	—	—	—	
Physical activity	Not active	−5.51	−8.72 to −2.29	−0.14	< .001
Sleeping difficulty	No (reference)	—	—	—	
Sleeping difficulty	Yes	−2.40	−5.49 to 0.69	−0.06	.127
HADS anxiety		−1.68	−2.11 to −1.25	−0.36	< .001
HADS depression		−1.11	−1.60 to −0.61	−0.22	< .001
Comorbidity count	0–5	0.42	−0.64 to 1.47	0.03	.438
HF duration	At current visit (reference)	—	—	—	
HF duration	<1 year	−3.25	−7.59 to 1.10	−0.07	.143
HF duration	>1 year	−1.01	−4.75 to 2.74	−0.02	.597
Prior HF admission	No (reference)	—	—	—	
Prior HF admission	Yes	−0.80	−3.94 to 2.34	−0.02	.618
R^2		0.560			
Adjusted R^2		0.532			
No. observations		476			

Note. B is unstandardized; confidence intervals and p values use HC3 covariance. Standardized β = B × SD(Xj)/SD(Y), including dummy columns. Reference categories are shown. HADS anxiety and depression were entered simultaneously.

S3. Complete model diagnostics

Table S3

Diagnostic statistics for the primary models

Outcome	n	BP χ^2 (p)	Shapiro W (p)	Skew	Excess kurt.	RESET F (p)	Rainbow (p)	Max adj. GVIF	|rstudent| >2 / >3	Cook >4/n / >1	High leverage
Physical	476	29.80 (.424)	0.991 (.007)	0.29	0.38	0.30 (.744)	0.85 (.882)	1.50	30 / 1	36 / 0	6
Mental	476	32.90 (.282)	0.993 (.020)	0.15	−0.32	0.71 (.495)	0.85 (.881)	1.50	18 / 1	20 / 0	6

Note. High leverage is defined as leverage > 2p/n. Cook screening thresholds were 4/n and 1.

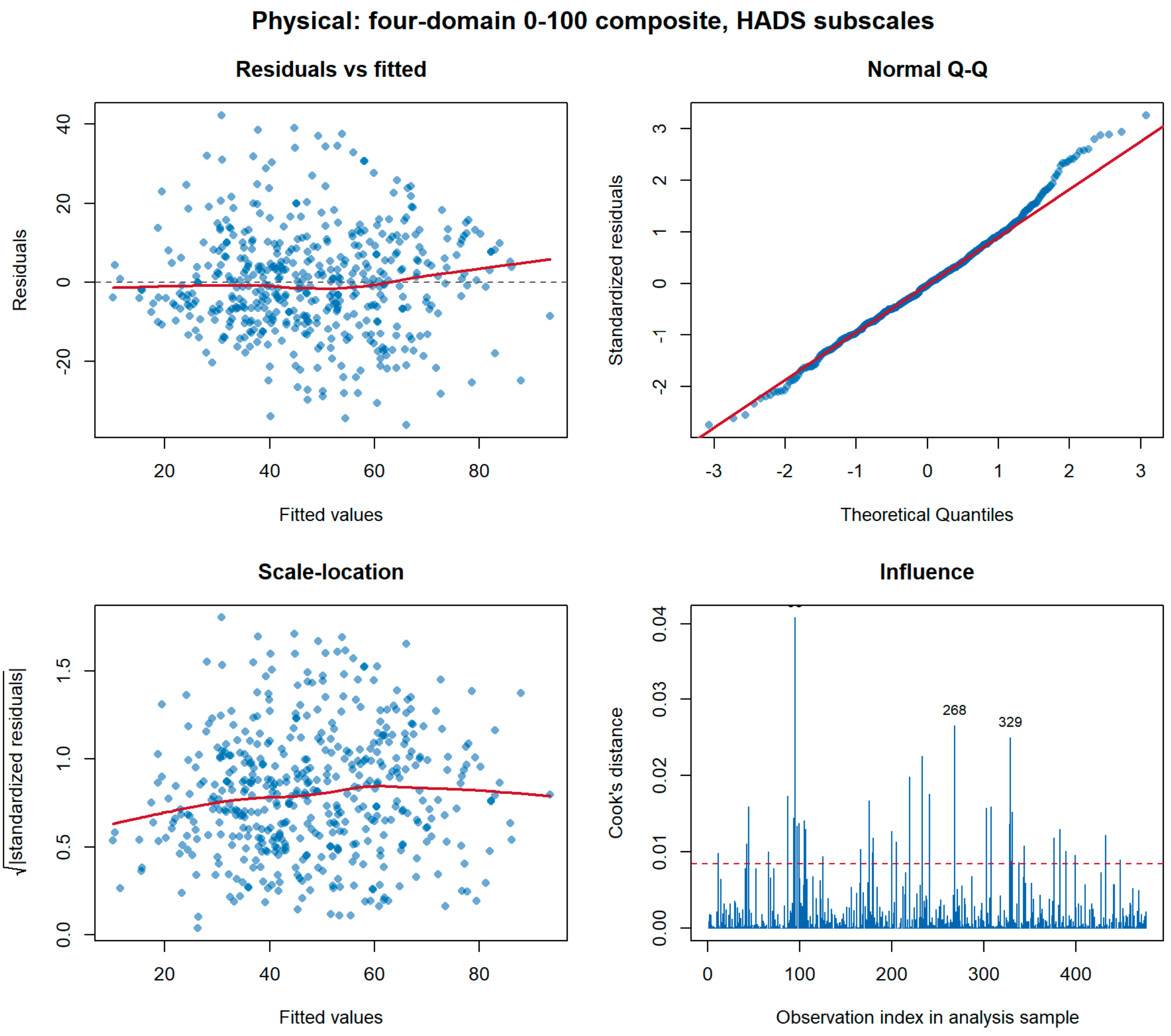

Figure S1. Diagnostic panels for the primary physical composite OLS model.

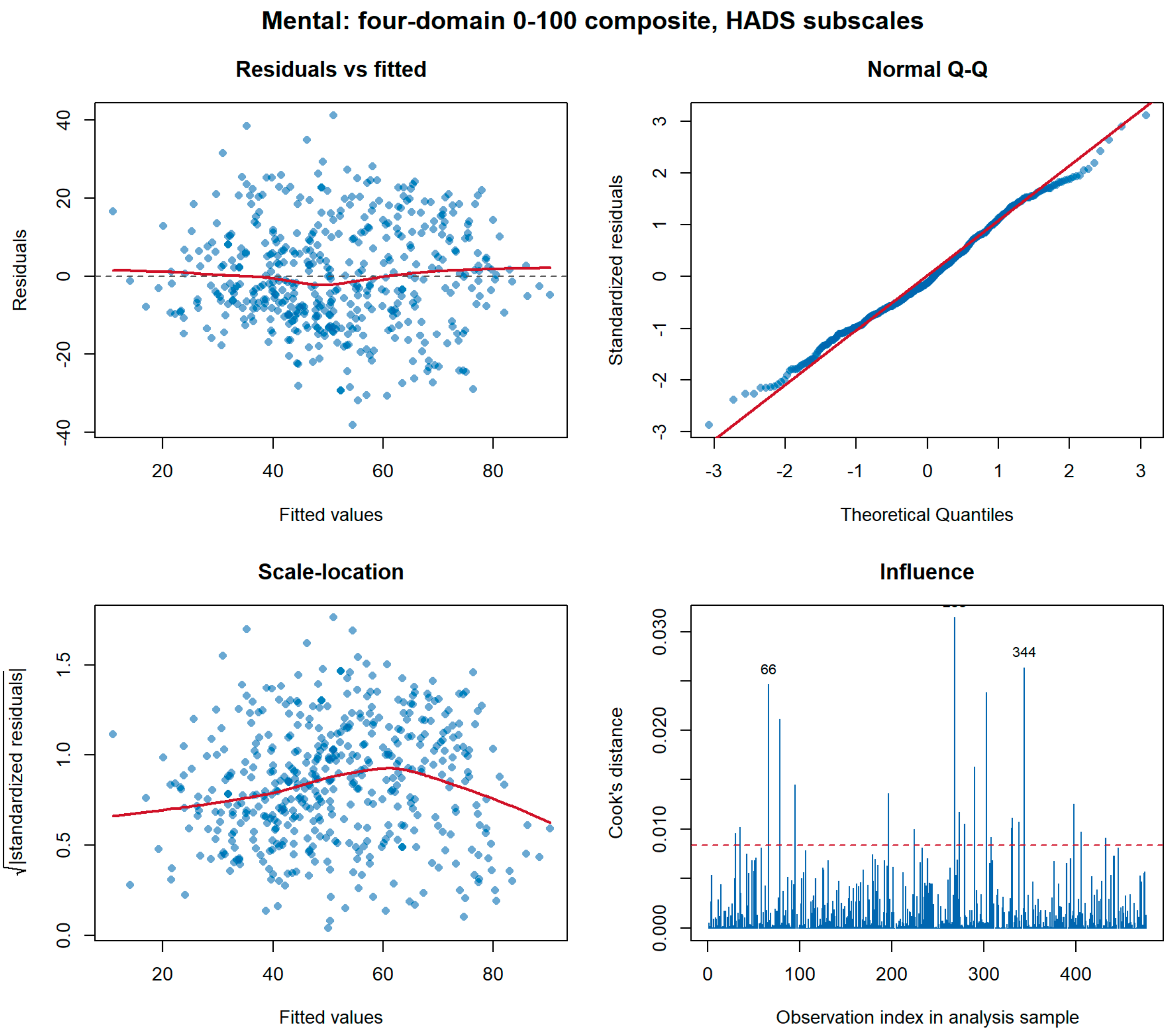

Figure S2. Diagnostic panels for the primary mental composite OLS model.

S4. Linearity assessments

Table S4

Separate quadratic-term tests for continuous predictors

Outcome	Predictor	Quadratic B	HC3 p	BH-adjusted p
Physical	Age, years	−0.0059	.270	.764
Physical	BMI, kg/m²	−0.0046	.426	.764
Physical	SCHFI: Maintenance	0.0016	.214	.764
Physical	SCHFI: Symptom perception	0.0010	.498	.764
Physical	SCHFI: Management	−0.0014	.385	.764
Physical	SCHFI: Self-efficacy	0.0001	.928	.974
Physical	AHFKT, %	0.0002	.950	.974
Physical	Monthly income, JOD	−0.0000	.974	.974
Physical	HADS anxiety	−0.0558	.084	.764
Physical	HADS depression	0.0195	.533	.764
Physical	Comorbidity count: 0–5	0.2040	.556	.764
Mental	Age, years	0.0044	.349	.766
Mental	BMI, kg/m²	−0.0033	.557	.766
Mental	SCHFI: Maintenance	0.0009	.464	.766
Mental	SCHFI: Symptom perception	−0.0009	.554	.766

Outcome	Predictor	Quadratic B	HC3 p	BH-adjusted p
Mental	SCHFI: Management	−0.0010	.498	.766
Mental	SCHFI: Self-efficacy	−0.0003	.775	.895
Mental	AHFKT, %	0.0062	.038	.420
Mental	Monthly income, JOD	0.0000	.281	.766
Mental	HADS anxiety	0.0073	.814	.895
Mental	HADS depression	0.0406	.179	.766
Mental	Comorbidity count: 0–5	−0.0015	.997	.997

Note. Each continuous predictor was tested in a separate augmented model. Benjamini–Hochberg adjustment was applied within outcome across 11 quadratic tests. No term remained significant after adjustment.

S5. Influence screening and exclusion refits

Table S5.1

Influence-exclusion sensitivity summary

Outcome	Full n	Flagged	Refit n	Max Cook	Max |ΔB|/outcome SD	Significance changes	Sign changes
Physical	476	36	440	0.041	0.072	5	1
Mental	476	20	456	0.031	0.177	3	2

Note. Flagged observations had Cook's distance > 4/n and were removed simultaneously for each refit. No observation had Cook's distance > 1.

Table S5.2

Full-sample and influence-exclusion coefficients for the physical composite

Term	Full B	Full p	Refit B	Refit p	ΔB/outcome SD	Sig. changed	Sign changed
Age, years	−0.03	.703	−0.01	.847	0.001	No	No
Sex: Female	−1.28	.557	−0.22	.899	0.051	No	No
BMI, kg/m²	−0.22	.051	−0.21	.018	0.000	Yes	No
NYHA class: II	−9.44	< .001	−9.70	< .001	−0.012	No	No
NYHA class: III	−14.63	< .001	−15.51	< .001	−0.042	No	No
EF category: HFmrEF (41–49%)	−5.94	.041	−7.42	< .001	−0.072	No	No
EF category: HFrEF (≤40%)	−5.45	.050	−6.90	.001	−0.070	Yes	No
SCHFI: Maintenance	0.01	.740	0.05	.132	0.002	No	No
SCHFI: Symptom perception	−0.08	.068	−0.09	.020	−0.000	Yes	No
SCHFI: Management	0.07	.192	0.05	.215	−0.001	No	No
SCHFI: Self-efficacy	0.08	.046	0.06	.039	−0.001	No	No
AHFKT, %	−0.07	.217	−0.02	.601	0.002	No	No
Education: Higher	2.22	.540	1.86	.467	−0.018	No	No
Education: Elementary	−0.54	.891	0.03	.992	0.028	No	Yes
Education: Secondary	1.45	.673	2.17	.356	0.035	No	No
Monthly income, JOD	0.00	.130	0.01	.003	0.000	Yes	No
Work status: Retired	−4.37	.012	−4.55	.002	−0.009	No	No
Work status: Not working	−1.79	.416	−2.48	.133	−0.033	No	No
Residency: Rural	−0.76	.596	−0.92	.435	−0.008	No	No
Marital status: Unmarried	2.77	.299	4.16	.046	0.067	Yes	No
Current smoker: Yes	−1.09	.473	−0.53	.669	0.027	No	No
Physical activity: Not active	−4.92	.002	−3.59	.003	0.065	No	No
Sleeping difficulty: Yes	−4.08	.013	−3.93	.002	0.007	No	No
Comorbidity count: 0–5	0.06	.913	0.43	.320	0.018	No	No
HF duration: <1 year	1.79	.441	0.87	.653	−0.045	No	No
HF duration: >1 year	3.36	.096	2.83	.073	−0.026	No	No
Prior HF admission: Yes	−1.88	.220	−2.04	.095	−0.008	No	No
HADS anxiety	−0.90	< .001	−1.01	< .001	−0.005	No	No
HADS depression	−1.40	< .001	−1.56	< .001	−0.008	No	No

Note. Significance changes indicate crossing the two-sided .05 threshold and are descriptive sensitivity results.

Table S5.3

Full-sample and influence-exclusion coefficients for the mental composite

Term	Full B	Full p	Refit B	Refit p	ΔB/outcome SD	Sig. changed	Sign changed
Age, years	0.03	.688	0.06	.429	0.001	No	No
Sex: Female	−0.97	.653	−1.35	.487	−0.018	No	No
BMI, kg/m²	0.08	.504	0.11	.310	0.002	No	No
NYHA class: II	−6.55	< .001	−6.43	< .001	0.006	No	No
NYHA class: III	−8.77	< .001	−8.41	< .001	0.018	No	No
EF category: HFmrEF (41–49%)	−1.81	.521	−0.38	.879	0.070	No	No
EF category: HFrEF (≤40%)	−2.58	.347	−1.37	.567	0.059	No	No
SCHFI: Maintenance	0.01	.890	0.04	.333	0.002	No	No
SCHFI: Symptom perception	−0.05	.313	−0.08	.034	−0.002	Yes	No
SCHFI: Management	−0.01	.804	0.02	.682	0.001	No	Yes
SCHFI: Self-efficacy	0.11	.010	0.09	.011	−0.001	No	No
AHFKT, %	0.04	.444	0.06	.244	0.001	No	No
Education: Higher	1.64	.645	4.86	.087	0.159	No	No
Education: Elementary	0.40	.920	3.11	.309	0.133	No	No
Education: Secondary	4.94	.140	8.54	.001	0.177	Yes	No
Monthly income, JOD	0.00	.229	0.00	.882	−0.000	No	No
Work status: Retired	−2.23	.193	−3.08	.051	−0.042	No	No
Work status: Not working	−1.54	.459	−3.61	.056	−0.102	No	No
Residency: Rural	0.05	.971	0.38	.771	0.016	No	No
Marital status: Unmarried	0.96	.748	0.13	.952	−0.041	No	No
Current smoker: Yes	−0.46	.761	−0.68	.625	−0.011	No	No
Physical activity: Not active	−5.51	< .001	−6.37	< .001	−0.042	No	No
Sleeping difficulty: Yes	−2.40	.127	−2.90	.048	−0.024	Yes	No
Comorbidity count: 0–5	0.42	.438	0.58	.231	0.008	No	No
HF duration: <1 year	−3.25	.143	−2.39	.240	0.042	No	No
HF duration: >1 year	−1.01	.597	−0.88	.622	0.006	No	No
Prior HF admission: Yes	−0.80	.618	0.28	.847	0.053	No	Yes
HADS anxiety	−1.68	< .001	−1.64	< .001	0.002	No	No
HADS depression	−1.11	< .001	−1.16	< .001	−0.003	No	No

Note. Significance changes indicate crossing the two-sided .05 threshold and are descriptive sensitivity results.

S6. EF-category comparisons

Table S6.1

Unadjusted composite scores by EF category in the full sample

Outcome	EF category	n	Mean (SD)	Median (IQR)
Physical	HFpEF (≥50%)	47	61.58 (20.03)	61.88 (44.69–77.50)

Outcome	EF category	n	Mean (SD)	Median (IQR)
Physical	HFmrEF (41–49%)	168	50.62 (21.09)	48.75 (34.69–65.00)
Physical	HFrEF (≤40%)	301	45.79 (20.52)	44.38 (32.50–58.75)
Mental	HFpEF (≥50%)	47	60.92 (19.54)	63.00 (48.44–77.71)
Mental	HFmrEF (41–49%)	168	53.87 (20.40)	49.83 (36.72–69.56)
Mental	HFrEF (≤40%)	301	49.36 (20.36)	46.96 (34.00–65.25)

Note. These full-sample descriptive results are independent of the multivariable model specification.

Table S6.2

Unadjusted EF omnibus tests

Outcome	n	HC3 F (df)	p	ω²
Physical	516	13.21 (2, 513)	< .001	0.044
Mental	516	8.02 (2, 513)	< .001	0.026

Table S6.3

Unadjusted Tukey-adjusted EF contrasts

Outcome	Contrast	Mean difference	Simultaneous 95% CI	Tukey p	Hedges g
Physical	HFmrEF (41–49%) minus HFpEF (≥50%)	−10.96	−18.89 to −3.03	.004	−0.52
Physical	HFrEF (≤40%) minus HFpEF (≥50%)	−15.79	−23.27 to −8.31	< .001	−0.77
Physical	HFrEF (≤40%) minus HFmrEF (41–49%)	−4.83	−9.57 to −0.09	.045	−0.23
Mental	HFmrEF (41–49%) minus HFpEF (≥50%)	−7.05	−14.77 to 0.67	.082	−0.35
Mental	HFrEF (≤40%) minus HFpEF (≥50%)	−11.56	−18.87 to −4.25	< .001	−0.57
Mental	HFrEF (≤40%) minus HFmrEF (41–49%)	−4.51	−9.14 to 0.11	.058	−0.22

Table S6.4

Adjusted EF omnibus tests from the primary models

Outcome	Numerator df	Denominator df	HC3 F	p
Physical	2	446	2.21	.111
Mental	2	446	0.49	.613

Table S6.5

Covariate-standardized marginal means by EF category

Outcome	EF category	Observed n	Adjusted mean	HC3 SE	95% CI
Physical	HFpEF (≥50%)	40	53.12	2.61	47.99 to 58.25
Physical	HFmrEF (41–49%)	153	47.17	1.30	44.62 to 49.73
Physical	HFrEF (≤40%)	283	47.66	0.84	46.01 to 49.32
Mental	HFpEF (≥50%)	40	53.57	2.56	48.54 to 58.59
Mental	HFmrEF (41–49%)	153	51.76	1.18	49.43 to 54.09
Mental	HFrEF (≤40%)	283	50.99	0.88	49.26 to 52.72

Note. Means average counterfactual predictions over the observed covariate distribution in the 476-participant model sample.

Table S6.6

Tukey-adjusted EF contrasts from the primary models

Outcome	Contrast	Adjusted difference	HC3 SE	Simultaneous 95% CI	Tukey p
Physical	HFmrEF (41–49%) minus HFpEF (≥50%)	−5.94	2.89	−12.75 to 0.86	.101
Physical	HFrEF (≤40%) minus HFpEF (≥50%)	−5.45	2.78	−11.99 to 1.08	.123
Physical	HFrEF (≤40%) minus HFmrEF (41–49%)	0.49	1.62	−3.32 to 4.30	.950
Mental	HFmrEF (41–49%) minus HFpEF (≥50%)	−1.81	2.81	−8.42 to 4.81	.797

Outcome	Contrast	Adjusted difference	HC3 SE	Simultaneous 95% CI	Tukey p
Mental	HFrEF (≤40%) minus HFpEF (≥50%)	−2.58	2.74	−9.02 to 3.86	.614
Mental	HFrEF (≤40%) minus HFmrEF (41–49%)	−0.77	1.52	−4.34 to 2.79	.867

Note. All three comparisons within each outcome were adjusted as one Tukey family using 446 residual degrees of freedom.

S7. Construct-overlap sensitivity

Table S7.1

Fit of construct-overlap sensitivity models

Model	n	Parameters	Residual df	R^2	Adjusted R^2
Physical model without NYHA	476	28	448	0.539	0.511
Mental model without HADS subscales	476	28	448	0.386	0.349

Note. The physical model excluded NYHA but retained both HADS subscales. The mental model excluded both HADS subscales.

Table S7.2

HC3 coefficients for the physical composite without NYHA

Term	B	95% CI	Standardized β	p
Age, years	−0.05	−0.23 to 0.13	−0.02	.558
Sex: Female	−2.60	−7.08 to 1.88	−0.05	.254
BMI, kg/m²	−0.21	−0.44 to 0.01	−0.06	.062
EF category: HFmrEF (41–49%)	−6.00	−11.89 to −0.11	−0.14	.046
EF category: HFrEF (≤40%)	−5.81	−11.53 to −0.09	−0.14	.046
SCHFI: Maintenance	0.04	−0.04 to 0.13	0.05	.312
SCHFI: Symptom perception	−0.11	−0.21 to −0.01	−0.11	.024
SCHFI: Management	0.06	−0.04 to 0.17	0.06	.230
SCHFI: Self-efficacy	0.09	0.00 to 0.17	0.10	.047
AHFKT, %	−0.07	−0.18 to 0.05	−0.04	.243
Education: Higher	1.68	−5.37 to 8.73	0.04	.639
Education: Elementary	0.64	−7.00 to 8.28	0.01	.869
Education: Secondary	1.38	−5.21 to 7.97	0.03	.681
Monthly income, JOD	0.01	−0.00 to 0.01	0.08	.065
Work status: Retired	−4.17	−7.77 to −0.56	−0.10	.023
Work status: Not working	−1.48	−6.02 to 3.06	−0.03	.523
Residency: Rural	−1.00	−3.94 to 1.94	−0.02	.506
Marital status: Unmarried	4.32	−1.42 to 10.06	0.05	.140
Current smoker: Yes	−1.44	−4.56 to 1.68	−0.03	.363
Physical activity: Not active	−6.15	−9.41 to −2.89	−0.15	< .001
Sleeping difficulty: Yes	−5.41	−8.72 to −2.10	−0.13	.001
Comorbidity count: 0–5	−0.05	−1.16 to 1.07	−0.00	.937
HF duration: <1 year	0.36	−4.30 to 5.01	0.01	.880
HF duration: >1 year	2.15	−1.93 to 6.24	0.05	.301
Prior HF admission: Yes	−2.19	−5.32 to 0.93	−0.05	.169
HADS anxiety	−0.90	−1.32 to −0.47	−0.19	< .001
HADS depression	−1.66	−2.14 to −1.19	−0.33	< .001

Note. The intercept is omitted. NYHA class was excluded, and both HADS subscales were retained.

Table S7.3

HC3 coefficients for the mental composite without the HADS subscales

Term	B	95% CI	Standardized β	p
Age, years	0.12	−0.07 to 0.30	0.06	.214
Sex: Female	−4.01	−9.10 to 1.09	−0.08	.123
BMI, kg/m²	0.14	−0.12 to 0.40	0.04	.298
NYHA class: II	−9.53	−13.88 to −5.17	−0.23	< .001
NYHA class: III	−13.11	−17.82 to −8.41	−0.30	< .001
EF category: HFmrEF (41–49%)	−2.50	−9.06 to 4.06	−0.06	.454
EF category: HFrEF (≤40%)	−3.23	−9.62 to 3.16	−0.08	.321
SCHFI: Maintenance	0.03	−0.07 to 0.14	0.03	.524
SCHFI: Symptom perception	−0.13	−0.23 to −0.03	−0.13	.013
SCHFI: Management	−0.05	−0.15 to 0.06	−0.05	.373
SCHFI: Self-efficacy	0.21	0.13 to 0.30	0.26	< .001
AHFKT, %	0.12	−0.01 to 0.24	0.07	.072
Education: Higher	5.64	−2.22 to 13.50	0.14	.159
Education: Elementary	4.23	−4.27 to 12.74	0.07	.328
Education: Secondary	8.10	0.83 to 15.37	0.20	.029
Monthly income, JOD	0.00	−0.00 to 0.01	0.06	.215
Work status: Retired	−3.10	−7.16 to 0.96	−0.08	.134
Work status: Not working	−1.96	−6.92 to 3.00	−0.04	.438
Residency: Rural	1.32	−1.94 to 4.58	0.03	.426
Marital status: Unmarried	−0.39	−7.72 to 6.94	−0.00	.917
Current smoker: Yes	−4.04	−7.48 to −0.60	−0.10	.021
Physical activity: Not active	−9.24	−12.88 to −5.60	−0.23	< .001
Sleeping difficulty: Yes	−7.12	−10.72 to −3.53	−0.18	< .001
Comorbidity count: 0–5	−0.85	−2.12 to 0.42	−0.06	.191
HF duration: <1 year	−0.47	−5.66 to 4.72	−0.01	.858
HF duration: >1 year	1.66	−2.76 to 6.08	0.04	.460
Prior HF admission: Yes	−0.73	−4.42 to 2.97	−0.02	.699

Note. The intercept is omitted, and both HADS subscales were excluded.

S8. Median-regression sensitivity

Table S8.1

Median-regression fit summary

Outcome	n	Parameters	Bootstrap replications	Pseudo R^2	Nonunique warning	Max solver difference/outcome SD
Physical	476	30	2000	0.395	No	0.0000
Mental	476	30	2000	0.365	No	0.0000

Note. Median regression used τ = .50 and 2,000 xy-pair bootstrap replications. Both HADS subscales were included.

Table S8.2

Median-regression coefficients for the physical composite

Term	B	Bootstrap SE	Bootstrap 95% CI	p
Age, years	0.06	0.10	−0.14 to 0.25	.571
Sex: Female	−0.97	2.66	−6.18 to 4.24	.715
BMI, kg/m²	−0.28	0.13	−0.54 to −0.02	.039
NYHA class: II	−9.58	2.22	−13.93 to −5.23	< .001
NYHA class: III	−16.77	2.66	−21.98 to −11.55	< .001
EF category: HFmrEF (41–49%)	−8.85	3.26	−15.23 to −2.47	.007
EF category: HFrEF (≤40%)	−7.25	3.03	−13.18 to −1.32	.017
SCHFI: Maintenance	0.03	0.05	−0.07 to 0.13	.562
SCHFI: Symptom perception	−0.08	0.05	−0.18 to 0.02	.118
SCHFI: Management	0.05	0.07	−0.08 to 0.18	.443
SCHFI: Self-efficacy	0.06	0.05	−0.04 to 0.16	.252
AHFKT, %	−0.07	0.06	−0.19 to 0.06	.287
Education: Higher	0.34	3.48	−6.48 to 7.16	.923
Education: Elementary	−2.62	3.91	−10.29 to 5.05	.504
Education: Secondary	0.05	3.29	−6.40 to 6.49	.988
Monthly income, JOD	0.00	0.00	−0.00 to 0.01	.251
Work status: Retired	−5.68	2.19	−9.98 to −1.39	.010
Work status: Not working	−3.45	2.37	−8.08 to 1.19	.146
Residency: Rural	−1.45	1.78	−4.94 to 2.04	.415
Marital status: Unmarried	4.02	2.94	−1.75 to 9.79	.172
Current smoker: Yes	0.09	1.86	−3.55 to 3.73	.963
Physical activity: Not active	−3.11	1.81	−6.67 to 0.45	.087
Sleeping difficulty: Yes	−4.40	2.06	−8.44 to −0.37	.033
Comorbidity count: 0–5	0.30	0.66	−1.00 to 1.60	.652
HF duration: <1 year	2.81	2.99	−3.05 to 8.68	.348
HF duration: >1 year	3.71	2.31	−0.82 to 8.24	.109
Prior HF admission: Yes	−1.76	1.76	−5.20 to 1.69	.318
HADS anxiety	−0.97	0.25	−1.45 to −0.48	< .001
HADS depression	−1.70	0.27	−2.23 to −1.17	< .001

Table S8.3

Median-regression coefficients for the mental composite

Term	B	Bootstrap SE	Bootstrap 95% CI	p
Age, years	0.04	0.12	−0.19 to 0.27	.743
Sex: Female	−1.51	3.31	−8.00 to 4.97	.647
BMI, kg/m²	0.00	0.16	−0.32 to 0.32	.999
NYHA class: II	−7.63	3.06	−13.62 to −1.64	.013
NYHA class: III	−9.03	3.17	−15.23 to −2.82	.005
EF category: HFmrEF (41–49%)	−0.37	4.34	−8.89 to 8.14	.932
EF category: HFrEF (≤40%)	−0.75	4.28	−9.15 to 7.65	.861
SCHFI: Maintenance	−0.01	0.06	−0.14 to 0.11	.861
SCHFI: Symptom perception	−0.10	0.06	−0.23 to 0.03	.125
SCHFI: Management	0.08	0.06	−0.04 to 0.20	.188
SCHFI: Self-efficacy	0.12	0.06	0.01 to 0.24	.030
AHFKT, %	0.06	0.08	−0.10 to 0.22	.488
Education: Higher	2.51	5.19	−7.67 to 12.69	.629
Education: Elementary	2.01	5.79	−9.33 to 13.35	.729
Education: Secondary	7.43	5.01	−2.40 to 17.25	.139
Monthly income, JOD	0.00	0.00	−0.00 to 0.01	.464
Work status: Retired	−1.85	2.45	−6.65 to 2.94	.449
Work status: Not working	−1.17	3.07	−7.19 to 4.86	.704
Residency: Rural	0.23	2.13	−3.94 to 4.40	.914
Marital status: Unmarried	1.81	3.55	−5.14 to 8.77	.609
Current smoker: Yes	−1.13	2.14	−5.33 to 3.06	.596
Physical activity: Not active	−6.11	2.21	−10.44 to −1.78	.006
Sleeping difficulty: Yes	−2.76	2.43	−7.52 to 2.01	.257
Comorbidity count: 0–5	−0.05	0.71	−1.44 to 1.33	.939
HF duration: <1 year	−3.20	3.42	−9.91 to 3.51	.350
HF duration: >1 year	−1.78	3.00	−7.67 to 4.10	.553
Prior HF admission: Yes	0.17	2.30	−4.35 to 4.68	.942
HADS anxiety	−1.86	0.30	−2.45 to −1.28	< .001
HADS depression	−0.92	0.36	−1.63 to −0.22	.010

Table S8.4

Median-regression omnibus tests

Outcome	Tested block	Wald χ^2	df	p
Physical	EF category	7.53	2	.023
Physical	Clinical-history covariates	3.07	4	.546
Mental	EF category	0.05	2	.975
Mental	Clinical-history covariates	0.89	4	.926

S9. US norm-based SF-36 Version 1 sensitivity

Table S9.1

Fit of US norm-based PCS and MCS sensitivity models

Outcome	n	Residual df	R^2	Adjusted R^2
PCS	476	446	0.464	0.429
MCS	476	446	0.453	0.417

Note. Only the outcome-scoring method changed; both HADS subscales and all other predictors were retained.

Table S9.2

HC3 coefficients for US norm-based PCS

Term	B	95% CI	Standardized β	p
Age, years	−0.05	−0.13 to 0.03	−0.05	.233
Sex: Female	−0.45	−2.55 to 1.65	−0.02	.673
BMI, kg/m²	−0.15	−0.27 to −0.03	−0.10	.015
NYHA class: II	−3.81	−5.57 to −2.05	−0.21	< .001
NYHA class: III	−6.81	−8.86 to −4.75	−0.34	< .001
EF category: HFmrEF (41–49%)	−2.29	−4.93 to 0.34	−0.12	.088
EF category: HFrEF (≤40%)	−2.10	−4.63 to 0.44	−0.11	.105
SCHFI: Maintenance	0.01	−0.04 to 0.05	0.01	.818
SCHFI: Symptom perception	−0.04	−0.09 to 0.01	−0.10	.080
SCHFI: Management	0.05	−0.00 to 0.10	0.11	.064
SCHFI: Self-efficacy	0.03	−0.01 to 0.07	0.07	.199
AHFKT, %	−0.05	−0.11 to 0.01	−0.07	.085
Education: Higher	1.34	−2.34 to 5.02	0.07	.475
Education: Elementary	0.57	−3.38 to 4.52	0.02	.776
Education: Secondary	0.40	−3.09 to 3.89	0.02	.822
Monthly income, JOD	0.00	−0.00 to 0.00	0.06	.258
Work status: Retired	−1.62	−3.37 to 0.14	−0.09	.071
Work status: Not working	−0.91	−3.13 to 1.32	−0.04	.423
Residency: Rural	−0.52	−1.97 to 0.93	−0.03	.479
Marital status: Unmarried	1.00	−1.80 to 3.81	0.03	.482
Current smoker: Yes	−0.35	−1.84 to 1.14	−0.02	.641
Physical activity: Not active	−2.03	−3.61 to −0.45	−0.11	.012
Sleeping difficulty: Yes	−2.13	−3.75 to −0.51	−0.12	.010
Comorbidity count: 0–5	−0.03	−0.58 to 0.52	−0.00	.910
HF duration: <1 year	1.78	−0.45 to 4.01	0.08	.118
HF duration: >1 year	2.17	0.17 to 4.17	0.12	.033
Prior HF admission: Yes	−0.83	−2.31 to 0.66	−0.04	.275
HADS anxiety	−0.13	−0.35 to 0.09	−0.06	.247
HADS depression	−0.59	−0.83 to −0.36	−0.26	< .001

Note. The intercept is omitted. Both HADS subscales were entered simultaneously.

Table S9.3

HC3 coefficients for US norm-based MCS

Term	B	95% CI	Standardized β	p
Age, years	0.05	−0.03 to 0.14	0.05	.226
Sex: Female	−0.45	−2.47 to 1.57	−0.02	.662
BMI, kg/m²	0.10	−0.02 to 0.22	0.07	.089
NYHA class: II	−1.63	−3.61 to 0.35	−0.09	.107
NYHA class: III	−1.49	−3.67 to 0.68	−0.07	.177
EF category: HFmrEF (41–49%)	−0.05	−2.74 to 2.65	−0.00	.972
EF category: HFrEF (≤40%)	−0.63	−3.27 to 2.01	−0.03	.641
SCHFI: Maintenance	0.00	−0.04 to 0.05	0.00	.992
SCHFI: Symptom perception	−0.00	−0.05 to 0.04	−0.01	.881
SCHFI: Management	−0.02	−0.07 to 0.02	−0.05	.277
SCHFI: Self-efficacy	0.05	0.01 to 0.09	0.13	.015
AHFKT, %	0.03	−0.02 to 0.09	0.05	.225
Education: Higher	0.35	−3.35 to 4.05	0.02	.852
Education: Elementary	−0.41	−4.39 to 3.57	−0.01	.840
Education: Secondary	2.22	−1.30 to 5.75	0.12	.216
Monthly income, JOD	0.00	−0.00 to 0.00	0.03	.485
Work status: Retired	−0.47	−2.20 to 1.27	−0.02	.595
Work status: Not working	−0.08	−2.22 to 2.07	−0.00	.944
Residency: Rural	0.28	−1.14 to 1.70	0.01	.696
Marital status: Unmarried	0.04	−3.07 to 3.14	0.00	.982
Current smoker: Yes	0.13	−1.36 to 1.61	0.01	.868
Physical activity: Not active	−1.80	−3.40 to −0.20	−0.10	.028
Sleeping difficulty: Yes	−0.30	−1.83 to 1.23	−0.02	.698
Comorbidity count: 0–5	0.21	−0.34 to 0.75	0.03	.459
HF duration: <1 year	−2.54	−4.74 to −0.35	−0.12	.023
HF duration: >1 year	−1.39	−3.32 to 0.55	−0.07	.161
Prior HF admission: Yes	−0.34	−1.87 to 1.20	−0.02	.668
HADS anxiety	−0.93	−1.17 to −0.70	−0.43	< .001
HADS depression	−0.36	−0.62 to −0.10	−0.16	.008

Note. The intercept is omitted. Both HADS subscales were entered simultaneously.
